# The impact of *Paenibacillus polymyxa* HY96-2 *luxS* on biofilm formation and control of tomato bacterial wilt

**DOI:** 10.1007/s00253-019-10162-0

**Published:** 2019-11-05

**Authors:** Jincui Yi, Daojing Zhang, Yuejuan Cheng, Jingjing Tan, Yuanchan Luo

**Affiliations:** State Key Laboratory of Bioreactor Engineering, East China University of Science and Technology, Shanghai, 200237 China

**Keywords:** *Paenibacillus polymyxa*, Quorum sensing, *luxS*, Biofilm formation, Bacterial wilt, Biocontrol efficacy

## Abstract

The focus of this study was to investigate the effects of *luxS*, a key regulatory gene of the autoinducer-2 (AI-2) quorum sensing (QS) system, on the biofilm formation and biocontrol efficacy against *Ralstonia solanacearum* by *Paenibacillus polymyxa* HY96-2. *luxS* mutants were constructed and assayed for biofilm formation of the wild-type (WT) strain and *luxS* mutants of *P. polymyxa* HY96-2 in vitro and in vivo*.* The results showed that *luxS* positively regulated the biofilm formation of HY96-2. Greenhouse experiments of tomato bacterial wilt found that from the early stage to late stage postinoculation, the biocontrol efficacy of the *luxS* deletion strain was the lowest with 50.70 ± 1.39% in the late stage. However, the *luxS* overexpression strain had the highest biocontrol efficacy with 75.66 ± 1.94% in the late stage. The complementation of *luxS* could restore the biocontrol efficacy of the *luxS* deletion strain with 69.84 ± 1.09% in the late stage, which was higher than that of the WT strain with 65.94 ± 2.73%. Therefore, we deduced that *luxS* could promote the biofilm formation of *P. polymyxa* HY96-2 and further promoted its biocontrol efficacy against *R. solanacearum*.

**Electronic supplementary material:**

The online version of this article (10.1007/s00253-019-10162-0) contains supplementary material, which is available to authorized users.

## Introduction

With the enhancement of people’s awareness of environmental protection and food safety, microbial pesticides have attracted more and more attention due to their non-toxicity, environmental friendliness, and safety toward humans and animals (Berg 2009). The microbial pesticide industry has become a “sunrise industry” in China in line with the Chinese government’s policy of “two reductions,” a policy that is focused on reducing the amounts of chemical pesticides and fertilizers used (Luo et al. 2018). The number of registered and commercially available biopesticides is growing sharply every year (http://www.icama.org.cn/fwb/index.jhtml). *Ralstonia solanacearum* is a devastating plant pathogen with a global distribution and an unusually wide host range, which could cause more than 200 plants throughout the world to be impacted by bacterial wilt (Genin and Boucher 2004). Bacterial wilt has been mainly controlled by chemical pesticides, but they are potentially harmful to the environment and have not been efficient in eradicating *R. solanacearum* (Marian et al. 2018). The use of microbial pesticides has become one of the important strategies to control plant bacterial wilt (Marian et al. 2018; Shen et al. 2017) because of their environmental friendliness, diversity of biocontrol mechanisms, and good control efficacy toward soil-borne diseases (Kalantari et al. 2018; Omomowo et al. 2018; Timmusk et al. 2019). As of December 2018, 52 microbial pesticides had been registered to control plant soil-borne diseases in China, of which 11 were registered to control plant bacterial wilt (http://www.icama.org.cn/fwb/index.jhtml).

*Paenibacillus polymyxa* HY96-2, which was isolated from the rhizosphere of tomato plants in the suburbs of Nanchang, Jiangxi Province, China, is a Gram-positive bacterium that has been shown to control a variety of plant diseases and promote plant growth (Fan et al. 2012; Luo et al. 2018; Xu et al. 2006b). As much as 1 billion CFU/g wettable powder of *P. polymyxa* HY96-2 has been developed and industrialized by our laboratory and a cooperative company as a microbial pesticide. This pesticide was registered in China in 2004 as the first microbial pesticide based on *Paenibacillus* for controlling plant bacterial wilt around the world (http://www.icama.org.cn/fwb/index.jhtml;https://www.epa.gov/tsca-inventory/list-substances-reported-under-tscainventory-notification-active-inactive-rule;http://www.ec.gc.ca/ese-ees/9F3909AA-3024-4BBD-AC9E-2EB681ED1BBD/ FSAR_Paenibacillus%20Polymyxa_EN.pdf). However, in order to provide a scientific basis for the field application technology of the pesticide with *P. polymyxa* HY96-2, further study of its biocontrol mechanism is needed. Preliminary studies suggested that *P. polymyxa* HY96-2 could control plant diseases through the mechanisms of colonization (biofilm formation), antagonism, and induced systemic resistance of plants in a similar manner as other microbial pesticides (Luo et al. 2018; Xu et al. 2006b). Among them, the most important factor for determining the biocontrol efficacy of a microbial pesticide is whether or not the biocontrol microorganisms can colonize well at the roots of the host plants (Ji et al. 2008; Li et al. 2012; Lugtenberg and Dekkers 1999). Biofilm formation around the roots of host plants is an important trait that has been linked to the colonization ability of biocontrol microorganisms (Haggag and Timmusk 2010; Li et al. 2012). Previous studies have shown that biofilm formation can improve the control efficacy of many biocontrol agents, including *Bacillus subtilis* (Bais et al. 2004) and *P. polymyxa* (Haggag and Timmusk 2010; Timmusk et al. 2009).

The colonization of biocontrol agents in the rhizosphere of host plant is strictly regulated by many environmental factors and the regulation system inside the bacteria. The quorum sensing (QS) system is one of the important factors that regulate the biofilm formation in many bacteria (Miller and Bassler 2001; Raafat et al. 2019). QS is a process by which bacteria monitor their population density and regulate gene expression by using secreted chemical signaling molecules called autoinducers (AI) (Balestrino et al. 2005; Miller and Bassler 2001). There are three typical quorum sensing systems that have been reported to date (Balestrino et al. 2005; Bassler 2002). Type 1 is a quorum sensing system mainly in Gram-negative bacteria that utilizes *N*-acyl-homoserine lactones (AHLs) as signal molecules (Galloway et al. 2011; Raafat et al. 2019; Whitehead et al. 2001). Type 2 quorum sensing system is mainly associated with Gram-positive bacteria and uses the autoinducing peptide (AIP) as a signal molecule (Tal-Gan et al. 2016). Type 3 quorum sensing system exists in both Gram-positive and Gram-negative bacteria and uses autoinducer-2 (AI-2) as a signal molecule (Bassler 2002; Galloway et al. 2012; Liu et al. 2019; Rezzonico et al. 2012). LuxS is the product of the *luxS* gene, and it catalyzes the conversion of *S*-ribosyl homocysteine (SRH) to homocysteine and 4,5-dihydroxy-2,3-pentanedione (DPD) (Balestrino et al. 2005). DPD then generates AI-2 spontaneously (Gu et al. 2018; Han and Lu 2009). As a result, *luxS* is a key regulatory gene of the AI-2-mediated quorum sensing system (Ma et al. 2017b).

Up until now, LuxS/AI-2 quorum sensing system research has mainly focused on human pathogens. It had been reported that *luxS* could affect the biofilm formation abilities of *Escherichia coli* (Niu et al. 2013), *Staphylococcus aureus* (Liu et al. 2019; Ma et al. 2017a), *Streptococcus oralis* (Rickard et al. 2006), *Streptococcus pneumoniae* (Vidal et al. 2011), *Streptococcus mutans* (Yoshida et al. 2005), and other species pathogens (Table 1), and most of their virulence could be affected by the biofilm formation regulated by *luxS* (Ali et al. 2018). Therefore, it could be speculated that the biofilm formation in probiotics would promote their biocontrol efficacy. There have been a few studies that have focused on the regulation of biofilm formation by *luxS* in probiotics. Sun et al. (2014) found that the overexpression of *luxS* in *Bifidobacterium longum* NCC2705 promoted its biofilm formation (Table 1). In *Bacillus*, sequencing results have shown that AI-2 was an important signaling molecule in QS systems of several species (such as *B. subtilis*, *Bacillus cereus*, *Bacillus thuringiensis*, *and Bacillus anthracis*), and *luxS* was the key regulatory gene of the QS systems in these organisms (Auger et al. 2006; Duanis-Assaf et al. 2015; Lombardia et al. 2006). Our previous study also showed that the QS system in *P. polymyxa* HY96-2 was mediated by AI-2, and its key regulatory gene was *luxS* (Luo et al. 2018).

**Table 1:** Summary of the effects of *luxS* on biofilm formation of some bacterial species

Species	G^+^/G^−^	Type	Regulation of *luxS* on the biofilm formation	The mechanism of *luxS* affecting biofilm formation	References
*Bifidobacterium breve* UCC2003	G^+^	Human probiotics	Positive regulation	Unclear	Christiaen et al. (2014)
*Bifidobacterium longum* NCC2705	G^+^	Human probiotics	Positive regulation	It may be a novel mechanism by which the AI-2 signal is transduced to affect QS independently of a LuxPQ or LsrB-type AI-2 receptor.	Sun et al. (2014)
*Bacillus subtilis* NCIB3610	G^+^	Contaminant bacterium in dairy industry	Positive regulation	Lactose-induced biofilm formation depends on the expression of the *tapA* and *epsA-O* operons, which are responsible for biofilm matrix production. Lactose enhanced the production of AI-2 affected not directly on the biofilm formation cascade. So, activation of biofilm formation via the QS system might be an additional regulatory mechanism.	Duanis-Assaf et al. (2015)
*Capnocytophaga ochracea*	G^−^	Human pathogens	Positive regulation	The attenuation of biofilm formation by a *luxS* deletion strain is likely caused by a defect in the activated methyl cycle rather than by a loss of AI-2.	Hosohama-Saito et al. (2016)
*Haemophilus influenzae*	G^−^	Human pathogens	Positive regulation	*luxS* impacts changes in lipooligosaccharides (LOS) glycoform populations that are essential for full biofilm maturation.	Armbruster et al. (2009)
*Paenibacillus polymyxa* HY96-2	G^+^	Biocontrol agent	Positive regulation	–	This study
*Streptococcus mutans*	G^+^	Human pathogens	Positive regulation	*luxS* regulates the glucosyltransferase genes that are required for sucrose-dependent biofilm formation.	Merritt et al. (2003)
*Streptococcus pneumoniae D39*	G^+^	Human pathogens	Positive regulation	LuxS regulates the transcript levels of *lytA*, which encodes an autolysin previously implicated in biofilms.	Vidal et al. (2011)
*Streptococcus suis*	G^+^	Animal pathogens	Positive regulation	*luxS* deletion affects biofilm formation via the LuxS-based signaling molecule (AI-2).	Wang et al. (2011)
*Bacillus cereus* ATCC 10987	G^+^	Human pathogens	Negative regulation	*luxS* repressed biofilm formation may be related to the *lsr-like* genes which were involved in uptake and processing of AI-2*.* The Lsr-like system is present in Gram-negative bacteria, but not found in any other sequenced Gram-positive bacterium except *Bacillus cereus*.	Auger et al. (2006)
*Listeria monocytogenes*	G^+^	Human pathogens	Negative regulation	The *luxS* gene may associate with repression of components required for attachment and biofilm formation.	Sela et al. (2006)
*Staphylococcus aureus*	G^+^	Human pathogens	Negative regulation	The LuxS/AI-2 QS system can regulate polysaccharide intercellular adhesion (PIA)-dependent biofilm formation via the repression of the *rbf* (a positive regulator of biofilm formation) expression.	Ma et al. (2017a)
*Staphylococcus epidermidis*	G^+^	Nosocomial pathogen	Negative regulation	*luxS* repressed biofilm formation by decreasing the transcription of intercellular adhesion operon (*ica*) genes and production of polysaccharide intercellular adhesin (PIA).	Xu et al. (2006a)

*G*^*+*^ Gram-positive, *G*^*−*^ Gram-negative

To the best of our knowledge, no research focusing on the biofilm formation and biocontrol efficacy of biocontrol agents, including *P. polymyxa*, regulated by *luxS* has been reported. Therefore, this study is the first to address the effect of *luxS* on the biofilm formation of *P. polymyxa*. Using the system of *P. polymyxa* HY96-2 wild-type strain and its *luxS* mutants against *R. solanacearum* in tomato plants, the impact of *luxS* on biocontrol efficacy of *P. polymyxa* HY96-2 was investigated. This study provides a scientific basis for the field application technology of the microbial pesticide derived from *P. polymyxa* HY96-2.

## Materials and methods

### Bacterial strains, plasmids, chemicals, media, and growth conditions

The strains and plasmids used in this study are listed in Supplementary Table S1, the primers are listed in Supplementary Table S2, and the chemicals are listed in Supplementary Table S3. *E. coli* DH5α cells (Woodcock et al. 1989) were cultured in LB medium at 37 °C with shaking at 200 rpm. *P. polymyxa* and its mutants were cultured in LB medium at 30 °C with shaking at 180 rpm. When necessary, antibiotics were used at the following concentrations: 100 μg/mL ampicillin, 25 μg/mL chloramphenicol, and 5 μg/mL erythromycin; the antibiotics were purchased from Saiguo Biotechnology Co., Ltd. (Guangzhou, Guangdong, China). *R. solanacearum* (ATCC11696) was activated using tetrazolium chloride (TZC) selective medium (Yang and Ho 1998) at 28 °C for 24 h, and the highly pathogenic colonies (pink colonies) were picked and suspended in sterile water to make an *R. solanacearum* suspension and spread on SPA plates (20 g sucrose, 5 g peptone, 0.5 g K_2_HPO_4_, 0.25 g MgSO_4_·7H_2_O and 15 g agar per liter, pH 7.2~7.4) for later use.

*P. polymyxa* HY96-2 was preserved in the China General Microbiological Culture Collection Center (CGMCC No. 0829). The accession number of the *P. polymyxa* HY96-2 complete genome sequence is CP025957, and the sequence number (locus tag) of the *luxS* gene of *P. polymyxa* HY96-2 is C1A50_RS02845.

### Construction of knockout plasmid pRN5101-*Cm*

Plasmid pRN5101-*Cm* was constructed for gene knockout by fusing the PCR products of the chloramphenicol resistance gene cloned from plasmid pDG1661 (Kim and Timmusk 2013) with the upstream and downstream fragments of the *luxS* genes amplified with the primers shown in Supplementary Table S2 by insertion into to the *Bam*HI/*Hin*dIII (Takara, Dalian, China) digested pRN5101 plasmid (Zhang et al. 2018) using the Hieff Clone™ Multi One Step Cloning Kit (Yeasen, Shanghai, China). The construct was then transferred into *E. coli* DH5α competent cells by heat shock. The *E. coli* DH5α strain with plasmid pRN5101-*Cm* was screened on LB agar with 100 μg/mL ampicillin and 5 μg/mL erythromycin. A total of 10 to 15 transformants were verified by PCR with primers pRN-F/pRN-R. The PCR products were sequenced by Shanghai Personal Biological Technology Co., Ltd. (Shanghai, China).

### Preparation of competent cells of *P. polymyxa* HY96-2 and electroporation

The method of preparing the competent cells of *P. polymyxa* HY96-2 was previously described by Zhang et al. (2011) with modifications. The *P. polymyxa* HY96-2 wild-type strain was activated on an LB plate for 24 h at 30 °C. A single colony was then inoculated into 50 mL LB broth and cultured at 30 °C with shaking at 180 rpm for 18 h. A 500-μL aliquot of culture broth of *P. polymyxa* HY96-2 was inoculated into 50 mL growth medium (LB broth + 0.5 M sorbitol), with shaking at 200 rpm and 30 °C until OD_600_ reached 0.6~0.8. The culture broth was cooled on ice for 10 min and then centrifuged at 10,000 rpm for 10 min at 4 °C to pellet the bacteria. The bacteria were washed with cooled sterile water one time and washed with cooled ETM buffer (0.5 M mannitol, 0.5 M sorbitol, 10% glycerol) three times (Zhang et al. 2011). The competent cells were then resuspended in 600 μL ETM buffer and stored at − 80 °C.

The pRN5101-*Cm* plasmid (2 μL, 120 ng/μL) and *P. polymyxa* HY96-2 competent cells (60 μL) were mixed on ice. The mixture was transferred into a 1-mm electric shock cuvette and cooled on ice for 10 min. The sample was pulsed with a voltage of 2.2 kV (capacitance of 25 μF and a resistance of 200 Ω) (Gao et al. 2014). After electroporation, 600 μL of resuscitation culture medium (LB medium + 0.5 M sorbitol + 0.38 M mannitol) was added for resuscitation, and the mixture was incubated at 30 °C with shaking at 180 rpm for 12 h. The mixture was then plated on LB agar with 25 μg/mL chloramphenicol and 5 μg/mL erythromycin and incubated at 30 °C for 24~48 h for selection. Transformants were verified by PCR with primers pRN-F/pRN-R, and PCR products were sequenced.

### Screening of *luxS* gene deletion strain *P. polymyxa* HY96-2-△*luxS*

Electroporated *P. polymyxa* HY96-2 cells with plasmid pRN5101 were cultured for 5 successive generations in LB broth with 25 μg/mL chloramphenicol at 41.5 °C. Cells from the 5th generation were plated on LB agar with 25 μg/mL chloramphenicol to screen the double crossover recombinants, which were verified by PCR (with primers *luxS*-F/*luxS*-R) and sequencing analysis.

### Construction of *luxS* gene expression vector pMA5-*luxS*

The *luxS* gene was cloned from *P. polymyxa* HY96-2 chromosomal DNA (with primer1121-F/1121-R) and was ligated into pMA5 (Liu and Du 2012) using *Bam*HI and *Nde*I (Takara, Dalian, China) cleavage sites with the Mut Express® MultiS Fast Mutagenesis Kit V2 (Vazyme, Shanghai, China). The ligation product was transformed into *E. coli* DH5α cells, which were screened on LB agar with 100 μg/mL ampicillin. Transformants were verified by PCR (with primers TY-F/TF-R) and sequencing analysis.

### Construction of the *luxS* gene complement strain *P. polymyxa* HY96-2-△*luxS::luxS*

The competent cells of strain *P. polymyxa* HY96-2-△*luxS* were prepared as *P. polymyxa* HY96-2. The pMA5-*luxS* plasmid was then electroporated into *P. polymyxa* HY96-2-△*luxS* competent cells. The electroporated cells were screened on LB agar with 100 μg/mL ampicillin. Transformants were verified by PCR (with primers TY-F/TF-R) and sequencing analysis. The verified positive mutants were the *luxS* gene complement strain, HY96-2-△*luxS::luxS.*

### Construction of *luxS* gene overexpression strain *P. polymyxa* HY96-2-*luxS*

Plasmid pMA5-*luxS* was electroporated into *P. polymyxa* HY96-2 competent cells, and the cells were screened on LB agar with 100 μg/mL ampicillin. The verified positive transformants were the *luxS* gene overexpression strain, *P. polymyxa* HY96-2-*luxS.*

### Analysis of the level of *luxS* gene expression of *P. polymyxa* HY96-2 wild-type strain and its mutants by quantitative PCR

The RNA extraction of the *P. polymyxa* HY96-2 wild-type strain and its mutants (HY96-2-△*luxS*, HY96-2**-**△*luxS::luxS*, HY96-2-*luxS*) was performed using TransZol UP Plus RNA Kit (Tiangen, Beijing, China). Purity and concentration of the RNA were determined using a microplate reader (SynergyMx, BioTek, Winooski, VT, USA). gDNA in total RNA was removed, and cDNA were synthesized using the TransScript One-Step gDNA Removal and cDNA Synthesis SuperMix (TransGen, Beijing, China). Purity and concentration of the cDNA were determined using a microplate reader, and the cDNA were diluted to 50 ng/uL with double-distilled H_2_O. Quantitative PCR (qPCR) experiments were performed using TransStart Top Green qPCR SuperMix (TransGen, Beijing, China) in 20 μL final volumes. PCR mixtures were prepared in nuclease-free water and contained 1×TransStart Top Green qPCR SuperMix, 0.2 μM of each primer (DL*luxS*-F/ DL*luxS*-R), and 50 ng of cDNA template. The 16S rRNA was selected as the reference gene, and the primers used were 27F/1492R (Supplementary Table S2). Amplifications were performed using a BIO-RAD CFX-96 real-time PCR system (Hercules, CA, USA) with the following thermal cycling protocol: 95 °C for 5 min; (95 °C for10 s, 56 °C for 10 s, 72 °C for 30 s) × 40 cycles and 72 °C for 5 min. Relative transcript abundance was calculated using the ΔΔCt method. The transcription of a given gene was calculated as the difference in qPCR threshold cycles (Ct). As one PCR cycle represents a twofold difference in template abundance, fold change values were calculated as 2^−ΔΔCt^. Three independent experiments were performed.

### Assay for biofilm formation in vitro

The *P. polymyxa* HY96-2 wild-type strain and its mutants were cultured in LB broth until OD_600_ reached 0.8. Aliquots of the different cell cultures (50 μL) were inoculated into 10 mL glass tubes with 5 mL LB broth and incubated unshaken at 30 °C for 2 to 8 days. After incubation, the cultured broth was carefully withdrawn and the test tubes were washed twice with sterile water. One milliliter of 1% (w/v) crystal violet was added to the test tubes and rolled in the test tubes to stain all biofilm. The test tubes were allowed to stand for 15 min at room temperature, and then, the solution was withdrawn and the test tubes were carefully washed five times with sterile water. Subsequently, 2.5 mL of acetone–ethanol (20:80, v/v) was added to dissolve the crystal violet binding to the biofilm (Yegorenkova et al. 2011). The absorbance (A_590_) of the solution was determined by a microplate reader (SynergyMx, BioTek, Winooski, VT, USA).

### Assay for biofilm formation in vivo

A total of 20 mL of cultured broth of the *P. polymyxa* HY96-2 wild-type strain and its mutants were poured into sterile plates. Tomato seedlings with heights of approximately 10 cm were pulled out of the sterilized soil, and their roots were washed with sterile water. The seedlings were then incubated in cell cultures of the HY96-2 wild-type strain and its mutants for 1 h; seedlings treated with sterile LB broth served as the control group. The treated plants were transferred to sterile nutrient solution and grown in a plant growth chamber (MGC-400, Yiheng, Shanghai, China) at 28 °C with a 16-h light regime. For the analysis, root segments with the length of 0.4~1 cm (Ren et al. 2012) were obtained and stored at − 80 °C after 2, 5, and 8 days of incubation. The root segments were immobilized at room temperature with 2.5% glutaraldehyde (preparation with 0.1 mol/L phosphate buffer) for 6 h. After that, the treated roots were washed 3 times with 0.1 mol/L phosphate buffer (pH 7.2) for 15 min (Thokchom et al. 2017). The treated samples were adhered to the sample table using a conductive adhesive. The colonization and biofilm formation of each strain on the roots of tomato plants were observed by cryo-scanning electron microscopy (S-3400N, Hitachi, Tokyo, Japan).

### Biocontrol efficacy of *P. polymyxa* HY96-2 wild-type strain and its mutants against *R. solanacearum*

Tomato seeds were sown in sterilized soil, and the seedlings with 3 to 4 leaves were transplanted into individual 10-cm pots 3 weeks later. Seedlings were cultivated under sufficient light and water in a greenhouse. The greenhouse experiment was carried out when the tomato seedlings grew to about 20 cm in height (at the 5 to 6 leaves stage of the seedlings, 8 to 10 days after transplanting), and six treatments were designed as follows: treatment 1, treated with water only (CK1); treatment 2, treated with *R. solanacearum* only (CK2); treatment 3, treated with *P. polymyxa* HY96-2 first and *R. solanacearum* later; treatment 4, treated with *P. polymyxa* HY96-2-△*luxS* first and *R. solanacearum* later; treatment 5, treated with *P. polymyxa* HY96-2**-***luxS* first and *R. solanacearum* later; and treatment 6, treated with *P. polymyxa* HY96-2-△*luxS::luxS* first and *R. solanacearum* later. On the 1st day of the experiment, the *P. polymyxa* HY96-2 WT or mutants were inoculated to the rhizosphere of the seedlings of treatment 3 to treatment 6, as well as treatment 1 and 2 just drenched with the same amount of water. On the 3rd day, the soil pathogen, *R. solanacearum*, was inoculated to the rhizosphere of the seedlings of all treatments except treatment 1. The drench dosages of *P. polymyxa* HY96-2 and *R. solanacearum* were 50 mL at 10^7^ CFU/mL. There were three biological replicates per treatment and 10 plants per replicate. The plants were incubated at 28 ± 1 °C in a greenhouse with relative humidity of 70% during the greenhouse experiment. The disease severity and control efficacy of each treatment were recorded and calculated on the 8th, 13th, and 18th day (5, 10, and 15 days after inoculation of *R. solanacearum*). The experiment was stopped when disease incidence reached 60% in CK2 (about at the 8 to 9 leaves stage of the seedlings).

The disease index (DI) was scored on the following 0–9 scale: 0, no visible symptoms; 1, one branch wilted at the top; 3, two branches wilted at the top; 5, three to four branches wilted; 7, only one branch is healthy; and 9, death of plant. The disease incidence, disease severity (DS), and biocontrol efficacy (BE) were calculated according to Li et al. (2017) and Wang et al. (2019) as follows:

$$ {D}_1=\frac{N_{\mathrm{I}}}{N}\times 100\% $$

where *D*_1_ is the disease incidence, *N*_I_ is the number of infected plants, and *N* is the total number of treated plants,

$$ \mathrm{DS}=\frac{\sum \left({N}_{\mathrm{i}}\times \mathrm{DI}\right)}{N_{\mathrm{d}}\times 9}\times 100\% $$

where *N*_i_ is the number of diseased plants of the corresponding disease index (DI), *N*_d_ is the total number of plants investigated, and the DI was recorded based on a scale of 0~9,

$$ \mathrm{BE}=\frac{{\mathrm{DS}}_{\mathrm{CK}}-{\mathrm{DS}}_{\mathrm{T}}}{{\mathrm{DS}}_{\mathrm{CK}}}\times 100\% $$

where DS_CK_ is the disease severity of CK2 and DS_T_ is the disease severity of plants treated with *P. polymyxa* HY96-2 wilt-type strain and its mutants.

### Statistical analyses

A minimum of three independent biological replicates were performed in all experiments. One-way analysis of variance (ANOVA) was carried out with SPSS (version 22.0, IBM, Armonk, NY, USA) and Dunnett’s multiple range test (*P* ≤ 0.05) for statistical analysis of all data.

## Results

### Construction and verification of *P. polymyxa* HY96-2 mutants

**Fig. 1:**
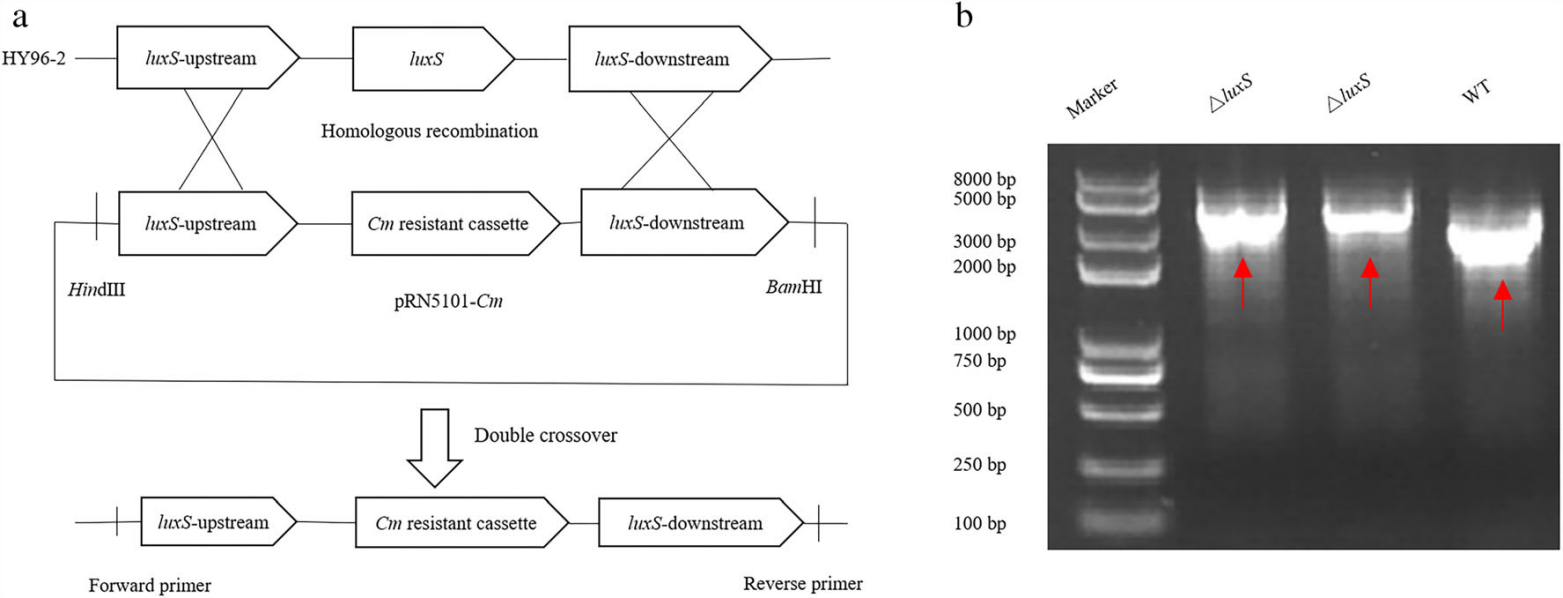
The construction of deletion mutants *P. polymyxa* HY96-2-△*luxS*. **a** A schematic diagram of double crossover recombinants obtained by homologous recombination. Cm, chloramphenicol. **b** PCR verification of the construction of deletion mutants *P. polymyxa* HY96-2-△*luxS*. The red arrows point to fragments cloned from transformants HY96-2-△*luxS* and HY96-2 wild-type strain with primers *luxS*-F and *luxS*-R. The sizes of the bands cloned from HY96-2-△*luxS* are 3770 bp, and the sizes of that from HY96-2 wild-type strain are 2967 bp

**Fig. 2:**
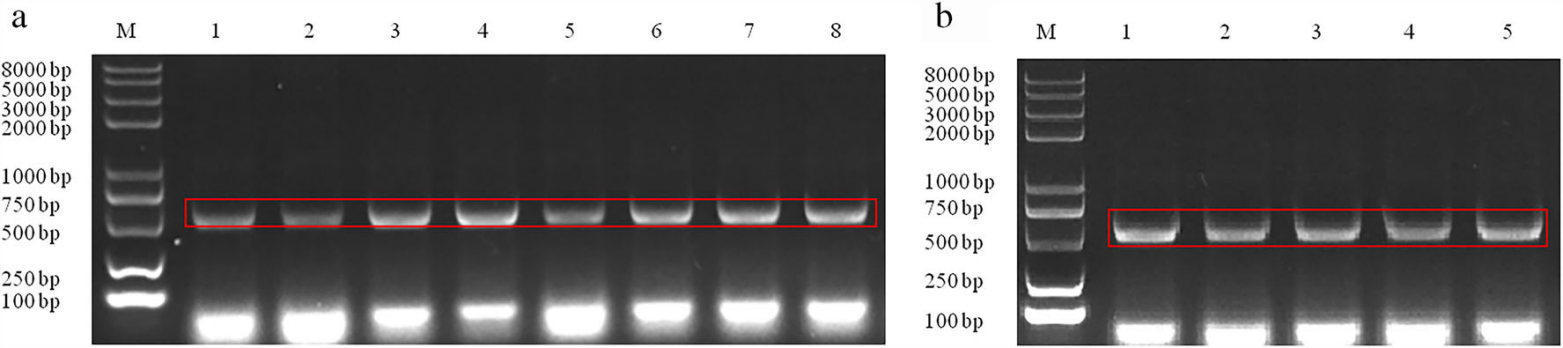
PCR verification of the construction of *luxS* complement mutants and overexpression mutants of *P. polymyxa* HY96-2. **a** PCR verification of the construction of complement mutants *P. polymyxa* HY96-2-△*luxS::luxS*. M, DNA marker; 1–8, PCR amplification of the *luxS* gene in positive transformants with primers TY-F and TY-R; the size of the expected bands is about 562 bp. **b** PCR verification of the construction of overexpression mutants *P. polymyxa* HY96-2-*luxS.* M, DNA marker; 1–5; PCR amplification of the *luxS* gene in positive transformants with primers TY-F and TY-R; the size of the bands is about 562 bp

Using homologous recombination, the *luxS* gene of *P. polymyxa* HY96-2 was replaced by a chloramphenicol resistance gene cassette to construct a *luxS* gene deletion strain (Fig. 1a). Then, the deletion mutant *P. polymyxa* HY96-2-△*luxS* obtained by electroporation with deletion plasmid pRN5101-*Cm* was verified by PCR (Fig. 1b). Furthermore, the *luxS* gene complement mutant, *P. polymyxa* HY96-2-△*luxS::luxS* (Fig. 2a), and *luxS* gene overexpression mutant, *P. polymyxa* HY96-2-*luxS* (Fig. 2b), obtained by electroporation with plasmid pMA5-*luxS* were verified by PCR. All plasmids and mutants were verified by PCR using genome-specific primers, and the PCR products were sequenced for further verification.

**Fig. 3:**
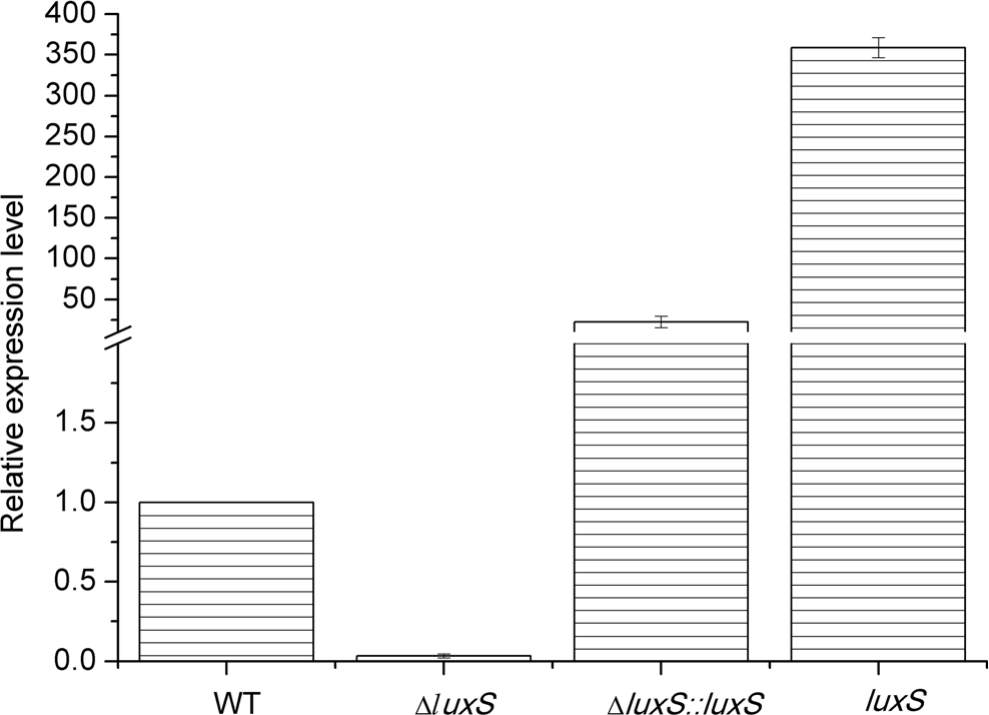
Analysis of the *luxS* gene expression level of *P. polymyxa* HY96-2 wild-type strain and its mutants by qPCR. All data represented the mean value of triplicate trials. WT, wild-type strain; △*luxS*, *luxS* deletion strain; *luxS*, *luxS* overexpression strain; △*luxS::luxS*, *luxS* complement strain. *P. polymyxa* HY96-2 wild-type strain was inoculated in LB broth, *P. polymyxa* HY96-2-△*luxS* was inoculated in LB broth with 25 μg/mL chloramphenicol, *P. polymyxa* HY96-2-△*luxS::luxS* and *P. polymyxa* HY96-2-*luxS* were inoculated in LB broth with 100 μg/mL ampicillin, and all of the stains were cultured at 30 °C with shaking at 180 rpm for 18 h

The expression levels of the *luxS* gene of the *P. polymyxa* HY96-2 wild-type strain and its mutants were compared by qPCR. The results (Fig. 3) showed that the *luxS* gene of *P. polymyxa* HY96-2-△*luxS* lacked significant expression, and the expression of the *luxS* gene from the complement strain *P. polymyxa* HY96-2-△*luxS::luxS* was 21.36-fold higher than that of the wild-type strain. The expression of the *luxS* gene from the overexpression strain *P. polymyxa* HY96-2-*luxS* was 358.27-fold higher than that of the wild-type strain.

### The *luxS* gene positively regulated the biofilm formation of *P. polymyxa* HY96-2 in vitro

**Fig. 4:**
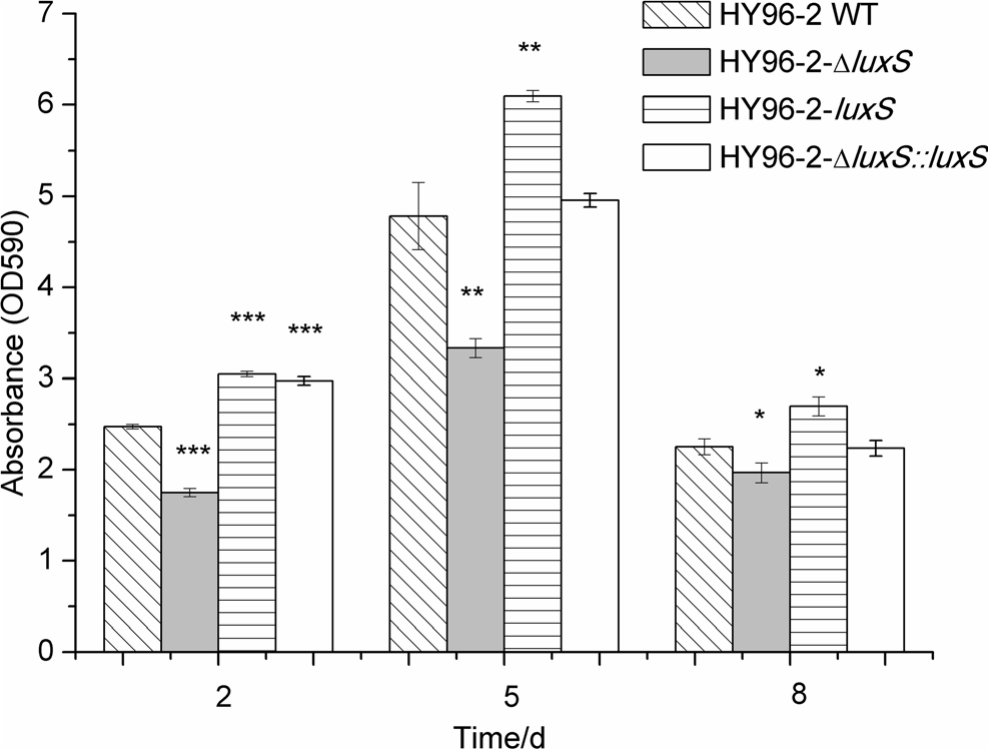
Assay for biofilm formation by *P. polymyxa* HY96-2 wild-type strain and its mutants in vitro. *P. polymyxa* HY96-2 wild-type strain was inoculated in LB broth, *P. polymyxa* HY96-2-△*luxS* was inoculated in LB broth with 25 μg/mL chloramphenicol, *P. polymyxa* HY96-2-△*luxS::luxS* and *P. polymyxa* HY96-2-*luxS* were inoculated in LB broth with 100 μg/mL ampicillin, and all of the stains were cultured unshaken at 30 °C. The biofilms were investigated at the 2nd, 5th, and 8th day postinoculation. One asterisk (*), two asterisks (**), and three asterisks (***) indicate significant difference between the treatments for a given time at *P* < 0.05, *P* < 0.01, and *P* < 0.001, respectively. All data represented the mean value of triplicate trials

An assay of biofilm formation in vitro was performed in glass tubes. It was found that the biofilm of the *P. polymyxa* HY96-2 wild-type strain was visible to the naked eye on the 2nd day after inoculation, and the biomass of the biofilm reached its maximum on the 5th day. The biofilm began to degrade on the 8th day, but the undegraded biofilm was stable for at least 20 days after inoculation (Supplementary Fig. S1). Therefore, the biofilm formation ability of the HY96-2 wild-type and its mutants was determined by crystal violet staining on the 2nd, 5th, and 8th day after inoculation. On the 2nd day after inoculation, compared with the wild-type *P. polymyxa* HY96-2 strain, the biofilm formation ability of the *P. polymyxa* HY96-2-△*luxS* strain decreased by 29.27%, and the biofilm formation ability of the *P. polymyxa* HY96-2-*luxS*, as well as the *P. polymyxa* HY96-2-△*luxS::luxS* strains, increased by 23.23% and 20.24%, respectively (Fig. 4). On the 5th day after inoculation, compared with the wild-type *P. polymyxa* HY96-2 strain, the biofilm-forming ability of the *P. polymyxa* HY96-2-△*luxS* strain decreased by 30.35%, and the biofilm formation ability of the *P. polymyxa* HY96-2-*luxS* strain increased by 27.46%, while the biofilm formation ability of the *P. polymyxa* HY96-2-△*luxS::luxS* strain showed no significant difference between that of the wild-type *P. polymyxa* HY96-2 strain (Fig. 4). On the 8th day after inoculation, compared with the wild-type *P. polymyxa* HY96-2 strain, the biofilm formation ability of the *P. polymyxa* HY96-2-△*luxS* strain decreased by 12.61%, and the biofilm formation ability of the *P. polymyxa* HY96-2-*luxS* strain increased by 19.75%, while the biofilm formation ability of the *P. polymyxa* HY96-2-△*luxS::luxS* strain still showed no significant difference between that of the wild-type *P. polymyxa* HY96-2 stain (Fig. 4). The images of biofilm stained by crystal violet also showed similar results (Supplementary Fig. S2). These results indicated that the deletion of *luxS* significantly reduced the biofilm formation ability of *P. polymyxa* HY96-2, while overexpression of *luxS* significantly improved its biofilm formation ability, and the complement of *luxS* gene could also significantly rescue its biofilm formation ability. Therefore, the AI-2 QS system regulated by *luxS* played an important role in biofilm formation of *P. polymyxa* HY96-2 in vitro, and *luxS* positively regulated the biofilm formation of this strain in vitro.

### The *luxS* gene positively regulated the biofilm formation of *P. polymyxa* HY96-2 in vivo

**Fig. 5:**
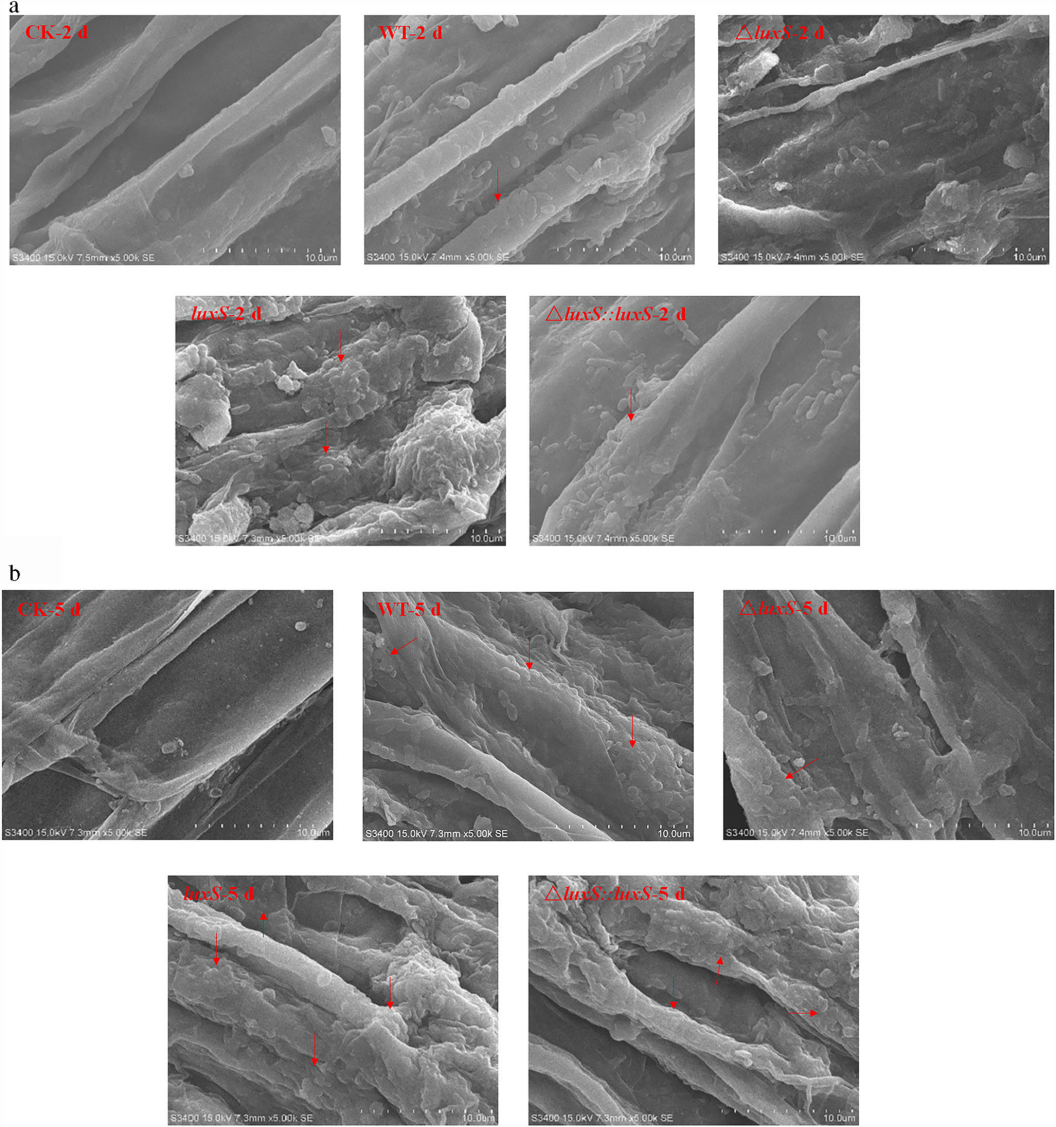

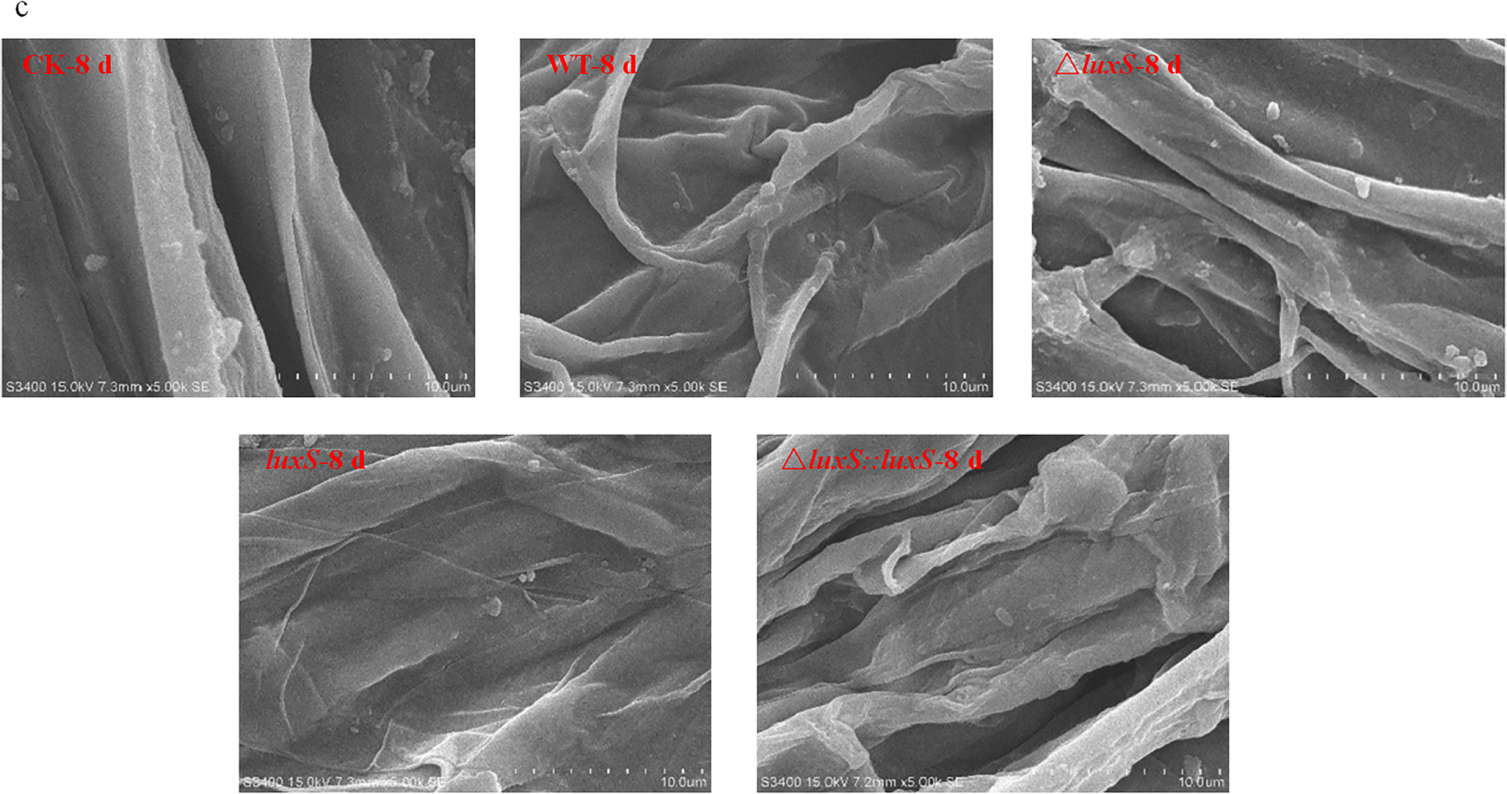
The biofilm formation by *P. polymyxa* HY96-2 wild-type strain and its mutants on the roots of tomato plants was observed by cryo-SEM at **a** 2 days, **b** 5 days, and **c** 8 days after inoculation. The red arrows indicate the spots where the HY96-2 cells aggregated obviously and formed the biofilm. CK, control; WT, wild-type strain; △*luxS*, *luxS* deletion strain; *luxS*, *luxS* overexpression strain; △*luxS::luxS*, *luxS* complement strain. All experiments were performed in triplicate, and a representative result was shown

The investigation of the colonization and biofilm formation of the *P. polymyxa* HY96-2 wild-type strain and its mutants on the roots of tomato plants was performed using cryo-SEM. The results showed that on the 2nd day after inoculation, HY96-2-△*luxS* had no obvious biofilm detected, while other strains began to form biofilms. The ability to colonize and form biofilm on the roots of tomato seedlings decreased in the strain order: *P. polymyxa* HY96-2-*luxS* > *P. polymyxa* HY96-2 wild-type≈*P. polymyxa* HY96-2-△*luxS::luxS* (Fig. 5a). On the 5th day after inoculation, more biofilm was formed and more bacteria were wrapped in them in all strains. The biofilm formed by strain *P. polymyxa* HY96-2-△*luxS* was reduced compared to that of the wild-type strain, while the biofilm formed by the *P. polymyxa* HY96-2-*luxS* and *P. polymyxa* HY96-2-△*luxS::luxS* strains was increased compared to that of the wild-type strain, and the amount of biofilm formed by strain *P. polymyxa* HY96-2-*luxS* was higher than that of strain *P. polymyxa* HY96-2-△*luxS::luxS* (Fig. 5b). Few bacteria or biofilms from any of the strains were observed on the 8th day after inoculation (Fig. 5c). These results suggested that the deletion of *luxS* reduced the colonization and biofilm formation ability of *P. polymyxa* HY96-2 on tomato roots, and the overexpression of *luxS* promoted these abilities; the complement of *luxS* could restore these abilities to at least levels similar to that of wild-type strain. These conclusions were consistent with the results observed in vitro. Therefore, the AI-2 QS system regulated by *luxS* also played an important role in the colonization and biofilm formation of *P. polymyxa* HY96-2 in vivo, and *luxS* also positively regulated the biofilm formation in vivo.

### The *luxS* gene positively regulated the biocontrol efficacy against *R. solanacearum* by *P. polymyxa* HY96-2

The results of the greenhouse experiments (Table 2) showed that the biocontrol efficacies of all treatments decreased with the increase of disease severity of CK2 (inoculated with *R. solanacearum* only) from the early stage (5 days after inoculation, the disease incidence of CK2 was 25.26%) to the late stage (15 days after inoculation, the disease incidence of CK2 was 61.35%) of tomato bacterial wilt. The biocontrol efficacy of strain *P. polymyxa* HY96-2-△*luxS* was the lowest during the whole test period, with 66.54 ± 5.31% in the early stage and 50.70 ± 1.39% in the late stage. These levels were significantly lower than that of wild-type strain with 82.37 ± 1.70% in the early stage and 65.94 ± 2.73% in the late stage. The biocontrol efficacy of strain *P. polymyxa* HY96-2-*luxS* was the highest during the whole test period with 100 ± 0.00% in the early stage and 75.66 ± 1.94% in the late stage, which was significantly higher than that of wild-type strain at the same disease stages. The biocontrol efficacy of strain *P. polymyxa* HY96-2-△*luxS::luxS* was similar to that of *P. polymyxa* HY96-2-*luxS* in the early stage of tomato bacterial wilt and similar to those of the wild-type strain at the peak and late stage of tomato bacterial wilt. These results indicated that the deletion of *luxS* significantly decreased the biocontrol efficacy against tomato bacterial wilt by *P. polymyxa* HY96-2, and the overexpression of *luxS* increased its biocontrol efficacy. The complement of *luxS* restored the biocontrol ability of the *luxS* deletion strain, *P. polymyxa* HY96-2-△*luxS*, to the level of wild-type strain or above. Therefore, the AI-2/*luxS* QS system played an important role in the biocontrol efficacy against tomato bacterial wilt in *P. polymyxa* HY96-2, and *luxS* positively regulated the biocontrol efficacy of the strain.

**Table 2:** The biocontrol efficacy against *R. solanacearum* by *P. polymyxa* HY96-2 wild-type strain and its mutants from the early stage to late stage of tomato bacterial wilt in greenhouse experiments

Time	CK1	CK2		HY96-2-WT		HY96-2-△*luxS*		HY96-2-*luxS*		HY96-2-△*luxS::luxS*	
	Disease incidence (%)	Disease incidence (%)	Disease severity (%)	Disease severity (%)	Control efficacy (%)	Disease severity (%)	Control efficacy (%)	Disease severity (%)	Control efficacy (%)	Disease severity (%)	Control efficacy (%)
Early stage (5 days postinoculation)	0.00	25.26 ± 4.40	6.82 ± 0.11	1.20 ± 0.13	82.37 ± 1.70^b^	2.28 ± 0.38	66.54 ± 5.31^c^	0.00 ± 0.00	100 ± 0.00^a^	0.00 ± 0.00	100 ± 0.00^a^
Peak stage (10 days postinoculation)	0.00	38.65 ± 1.03	16.73 ± 0.98	4.35 ± 0.13	73.90 ± 1.73^b^	7.31 ± 0.73	56.37 ± 2.02^c^	2.59 ± 0.26	84.52 ± 0.98^a^	4.35 ± 0.92	74.20 ± 3.95^b^
Late stage (15 days postinoculation)	0.00	61.35 ± 1.03	24.75 ± 1.81	8.43 ± 0.91	65.94 ± 2.73^b^	12.19 ± 0.79	50.70 ± 1.39^c^	6.02 ± 0.65	75.66 ± 1.94^a^	7.47 ± 0.68	69.84 ± 1.09^ab^

The data presented the mean value of three biological replicates (10 plants per replicate). Values with different lowercase letters in the same row showed significant differences at *P* < 0.05 (*n* = 3)

## Discussion

It has been reported that the *luxS* gene, which is a key regulatory gene of the AI-2-mediated quorum sensing (QS) system, affected the biomass and morphology of biofilms formed by some bacterial species. For some species, *luxS* expression positively regulated their biofilm formation. These species (Table 1) included Gram-negative human pathogens *Haemophilus influenzae* (Armbruster et al. 2009) and *Capnocytophaga ochracea* (Hosohama-Saito et al. 2016), Gram-positive human pathogens *S. mutans* (Yoshida et al. 2005) and *S. pneumoniae* (Vidal et al. 2011), Gram-positive animal pathogens *Streptococcus suis* (Wang et al. 2011), and Gram-positive human probiotics *B. longum* (Sun et al. 2014), *Bifidobacterium breve* (Christiaen et al. 2014), and *B. subtilis*, which is a contaminant bacterium in the dairy industry (Duanis-Assaf et al. 2015). However, *luxS* negatively regulated the biofilm formation in other species (Table 1), including the Gram-positive human pathogens *S. aureus* (Ma et al. 2017a), *Staphylococcus epidermidis* (Xu et al. 2006a), *Listeria monocytogenes* (Sela et al. 2006), and *B. cereus* (Auger et al. 2006). In addition, *luxS* also showed marked differences in biofilm structure between the wild-type strain and the *luxS* mutants in *S. mutans*. The *luxS* deletion strain of *S. mutans* adopted a much more granular biofilm, rather than the relatively smooth biofilm seen in the wild-type strain (Merritt et al. 2003). The biofilm of the *S. suis* wild-type strain was multi-layered with more extracellular matrix, but the biofilm formed by the *luxS* deletion strain was less dense with less extracellular matrix (Wang et al. 2011). The *luxS* deletion strain of *S. epidermidis* generated a more compact and thicker biofilm than that of the wild-type strain (Xu et al. 2006a).

The impact of *luxS* on the biofilm formation varies for different species of *Bacillus*, which might be related to the different regulatory mechanisms of *luxS* in different *Bacillus* species. *luxS* positively regulated the biofilm formation of *B. subtilis* NCIB3610, which is a contaminant bacterium in the dairy industry (Table 1). Duanis-Assaf et al. (2015) found that lactose, the primary sugar in milk, might induce the biofilm formation of *B. subtilis* by promoting the expression of the *tapA* and *epsA-O* operons, which are responsible for biofilm matrix production. In this case, it seemed that lactose enhanced the production of AI-2 rather than the biofilm formation cascade (Duanis-Assaf et al. 2015). The activation of biofilm formation via the QS system might be an additional regulatory mechanism which enabled fine tuning of the biofilm formation pathway (Duanis-Assaf et al. 2015). On the contrary, *luxS* negatively regulated the biofilm formation of *B. cereus*, which is a causative agent of food-borne diseases (Table 1). Auger et al. (2006) found that the regulatory mechanism of *luxS* on biofilm formation might be related to the *lsr-*like genes in *B. cereus* ATCC 10987. The Lsr-like system could be responsible for AI-2 uptake and processing (Auger et al. 2006). It was worth noting that the Lsr-like system was recently found in Gram-negative bacteria, such as *Salmonella typhimurium* and *E. coli*, but not in any other sequenced Gram-positive bacteria, including *B. subtilis*, *Bacillus halodurans*, or *Listeria* spp. (except *B. cereus*) (Auger et al. 2006).

As a biocontrol agent, *P. polymyxa* formed biofilms around the roots of plants (Timmusk et al. 2005; Yegorenkova et al. 2013) and *luxS* was reported as the key regulatory gene in its QS system (Luo et al. 2018). However, there have been no reports to date on *luxS* regulating the biofilm formation of biocontrol agents, including *P. polymyxa*. Therefore, in this study, the effect of *luxS* on biofilm formation of *P. polymyxa* HY96-2 wild type and its *luxS* mutants, *P. polymyxa* HY96-2-△*luxS*, *P. polymyxa* HY96-2-△*luxS::luxS*, and *P. polymyxa* HY96-2-*luxS*, was reported. The results in vitro and in vivo showed that the deletion of *luxS* significantly reduced the biofilm formation ability of strain HY96-2, while overexpression of *luxS* significantly improved its biofilm formation ability and the complement of *luxS* gene restored its biofilm formation ability. In addition, *luxS* did not change the morphology of the biofilm formed by *P. polymyxa* HY96-2. Both the wild-type strain and the *luxS* mutants of *P. polymyxa* HY96-2 formed white and viscous biofilms on the wall of test tubes and smooth and transparent biofilms on the roots of tomato plants.

Among *Bacillus*, the relatively clear biofilm formation pathways of *B. subtilis* have been reported, and these pathways mainly included the Spo0A regulatory pathway, the SlrR-SinR epigenetic switch, and the DegS-DegU two-component system (Vlamakis et al. 2013). *B. cereus* was also reported to have a regulation of biofilm formation through the Spo0A pathway (Xu et al. 2017). Our previous study showed that the possible pathways of biofilm formation in *P. polymyxa* included the Spo0A regulatory pathway and the DegS-DegU pathway (Luo et al. 2018). However, slight differences between the Spo0A regulatory pathways of *P. polymyxa* and *B. subtilis* were detected. Compared with *B. subtilis*, *P. polymyxa* lacked a mediator Spo0B, which transferred the phosphate group from Spo0F to Spo0A (Luo et al. 2018). Considering the completely different regulatory mechanisms of *luxS* on the biofilm formation of *B. subtilis* and *B. cereus* (Table 1), as well as the differences between *P. polymyxa* and *B. subtilis* in biofilm formation pathways, the question of whether the mechanism of *luxS* affecting biofilm formation in *P. polymyxa* HY96-2 is the same as that in *B. subtilis* requires further study.

In the evaluation of the biofilm formation ability in vivo, 8 days after inoculation, there were few biofilms detected in the treatment of *P. polymyxa* HY96-2 wild-type or in the other treatments of its mutants. It was speculated that this might be due to the fact that tomato seedlings were cultured in liquid soilless culture with limited nutrition. On the 8th day, due to insufficient nutrition, the growth of tomato seedlings was weak, the bacteria attached to its roots were not provided sufficient nutrition, and the biofilm of the strains began to degrade. The bacteria then broke away from the root of the tomato seedlings with the degradation of unstable biofilm. Similarly, the colonization investigation of *P. polymyxa* C5 on tobacco roots also showed that the cell density began to decrease 9 days after inoculation (Ren et al. 2012). However, the cell density in that study seemed to have decreased less than that of *P. polymyxa* HY96-2 because tobacco plants were cultured in plastic cups with 300 g of soil, which provided more nutrition.

Biofilms are a microbial community attached to the surface of an object (Mah and O’Toole 2001; O'Toole et al. 2000). Microorganisms in biofilms live in their own extracellular polymers (EPS), which are mainly composed of polysaccharides, proteins, nucleic acids, and lipids (Flemming and Wingender 2010). Previous studies have shown that biofilms formed by some biocontrol agents could facilitate their biocontrol efficacy especially against the soil-borne diseases. Bais et al. (2004) reported that biocontrol of *B. subtilis* against infection of *Arabidopsis* roots by *Pseudomonas syringae* was facilitated by biofilm formation. Klein and Kupper (2018) found that biofilm formed by the fungus *Aureobasidium pullulans* ACBL-77 enhanced the ability of biocontrol efficacy against sour rot in citrus. The biofilm formation by *P. polymyxa* also showed a significant impact on the improvement of biocontrol efficacy. Timmusk et al. (2009) reported that *P. polymyxa* strains with better biofilm formation ability had higher biocontrol efficacy against *Phytophthora palmivora* and *Pythium aphanidermatum* in *Arabidopsis thaliana*. Haggag and Timmusk (2010) found that colonization of peanut roots by the biofilm-forming strain *P. polymyxa* initiated biocontrol against crown rot disease. Ren et al. (2012) suggested that biofilm formation of the *P. polymyxa* C5 strain in tobacco roots is one of the mechanisms used to protect tobacco from fungal infection. It has long been considered that the function of biofilm formation of biocontrol agents is to resist pathogens invading plant roots and restricts colonization sites and nutrition in the rhizosphere of plants; the impact of this is to limit the population of the pathogens and control disease (Bais et al. 2004). The latest research of Timmusk et al. (2019) showed that the *P. polymyxa* A26 antagonistic activity against Fusarium Head Blight caused by *Fusarium graminearum* was positively correlated with d-glucuronate content (and not a common non-ribosomal antibiotic lipopeptide) in biofilm extracellular polysaccharides. *P. polymyxa* HY96-2 effectively resisted the invasion of *R. solanacearum*, as was verified by our previous studies (Xu et al. 2006b). However, further studies are needed to determine whether the biofilm matrix of *P. polymyxa* HY96-2 contains some substance which can inhibit the growth of pathogenic bacteria and improve its biocontrol efficacy against bacterial wilt.

It has been reported that *luxS*, a key regulator of AI-2 QS, can affect the virulence or biocontrol efficacy of the strains by affecting their biofilm formation abilities. For example, the *luxS* deletion mutant of *E. coli* 107/86 exhibited reduced biofilm formation and decreased pathogenicity (Yang et al. 2014). The *luxS* deletion mutant of *B. breve* UCC2003 showed decreased colonization ability in the intestinal tract of mice and *Caenorhabditis elegans*, which resulted in a worsened effect on preventing *C. elegans* from being infected by *Salmonella* than that of the wild-type strain (Christiaen et al. 2014). At present, there is no reported research about how *luxS* affects the biocontrol efficacy of biocontrol agents, including *P. polymyxa*. However, other QS systems have been suggested to regulate biofilm formation and affect biocontrol efficacy in Gram-negative biocontrol agents. The PcoI-PcoR QS system (LuxR–LuxI family) found in *Pseudomonas fluorescens* 2P24 showed a significant effect on its biofilm formation and biocontrol efficacy. The *pcoI* deletion mutant of strain 2P24 significantly reduced the biofilm formation, as well as colonization, on wheat rhizosphere, and then affected its biocontrol ability against wheat take-all (Wei and Zhang 2006).

In this study, the effect of *luxS* on biocontrol efficacy against *R. solanacearum* by *P. polymyxa* was investigated with *P. polymyxa* HY96-2 wild-type and its mutants. The results (Table 2) indicated that *luxS* positively regulated the biocontrol efficacy of strain HY96-2. In *P. polymyxa* HY96-2, the impact of *luxS* on biofilm formation was consistent with its effect on biocontrol efficacy against *R. solanacearum*. Therefore, we concluded that *luxS* improved the biocontrol efficacy of *P. polymyxa* HY96-2 by promoting its biofilm formation ability. More biofilm was formed by the overexpression strain, *P. polymyxa* HY96-2-*luxS*, and encapsulated more bacteria in it, which occupied more physiological sites on the roots of tomato plants to prevent the invasion of *R. solanacearum*. On the other hand, more cells of strain HY96-2 located in the rhizosphere of tomato plant would consume more nutrients, so less nutrition would be available for *R. solanacearum*, which would limit its population and the infecting probability of *R. solanacearum* on tomato plants. On the contrary, less biofilm formed by the deletion strain, *P. polymyxa* HY96-2-△*luxS*, would result in more physiological sites exposed on the roots of tomato plants to *R. solanacearum*, and more nutrition would be available to *R. solanacearum*, which would increase the incidence of bacterial wilt in tomato plants.

In summary, *luxS* played an important role in *P. polymyxa* HY96-2 biofilm formation and biocontrol efficacy against *R. solanacearum*. According to our results, it could be deduced that *luxS* improved the biofilm formation of *P. polymyxa* HY96-2 and then further promoted its biocontrol efficacy against *R. solanacearum*. This result could be used to guide the development of field application technology of the microbial pesticides with *P. polymyxa* HY96-2 and provide a scientific basis for improving the field biocontrol efficacy of industrialized *P. polymyxa* HY96-2 products. These results could also provide a reference for investigating the effect of QS systems on the biocontrol efficacy of other biocontrol agents.

## Electronic supplementary material

ESM 1 (PDF 252 kb)
