# Supplementary material for: The impact of Paenibacillus polymyxa HY96-2 luxS on biofilm formation and control of tomato bacterial wilt
Source: Appl Microbiol Biotechnol. 2019 Nov 5;103(23):9643–57. doi: 10.1007/s00253-019-10162-0 (PMC6867978; doi:10.1007/s00253-019-10162-0)
Supplement: Supplementary file 1 — (PDF 252 kb) [file 253_2019_10162_MOESM1_ESM.pdf]

Supplementary material

Article title: The impact of *Paenibacillus polymyxa* HY96-2 *luxS* on biofilm formation and control of tomato bacterial wilt

Journal name: Applied Microbiology and Biotechnology

Author: Jincui Yi[1] • Daojing Zhang[1] •Yuejuan Cheng[1] • Jingjing Tan[1] • Yuanchan Luo[1*]

Affiliation: [1] State Key Laboratory of Bioreactor Engineering, East China University of Science and Technology, Shanghai 200237, China

e-mail address: *Address correspondence to Yuanchan Luo, luoyuanc@ecust.edu.cn

Phone and Fax: 86-21-64252104

1. Supplementary tables

Table S1 Strains and plasmids used in this study

Table S2 Primers used in this study

Table S3 Chemicals used in this study

2. Supplementary figures

Fig. S1 Images of biofilms of *Paenibacillus polymyxa* HY96-2 and its mutants attached glass tubes on the 20th day post inoculation

Fig. S2 Images of biofilms of *P. polymyxa* HY96-2 and its mutants attached glass tubes stained by crystal violet

1. Supplementary tables

Table S1 Strains and plasmids used in this study

Strain or plasmid	Relevant characteristics	Reference or source
**Strains**		
Escherichia coli DH5α	Replication of plasmid pMA5-*luxS* and pRN5101-*Cm*	Woodcock et al. (1989)
Paenibacillus polymyxa HY96-2	Wild type, G^+, isolated from the rhizosphere of tomato plants in the suburbs of Nanchang, Jiangxi Province, China, and preserved in the China General Microbiological Culture Collection Center (CGMCC No. 0829), the accession number of its complete genome sequence is CP025957	China General Microbiological Culture Collection Center, Beijing, China
P. polymyxa HY96-2-△*luxS*	*P. polymyx*a HY96-2 *luxS* gene deletion strain, *luxS* gene was replaced by chloramphenicol resistance gene from plasmid pDG1661, Amp^r, Cm^r	This study
P. polymyxa HY96-2-△*luxS::luxS*	*P. polymyx*a HY96-2 *luxS* gene complement strain, *P. polymyxa* HY96-2-△*luxS* transformed with plasmid pMA5-*luxS*, Amp^r, Kan^r	This study
P. polymyxa HY96-2-*luxS*	*P. polymyx*a HY96-2 *luxS* gene over expression strain, *P. polymyxa* HY96-2 WT transformed with plasmid pMA5-*luxS*, Amp^r, Kan^r	This study
Ralstonia solanacearum ATCC11696	Isolated from tomato plant, G^-, pathogen of Bacterial wilt	ATCC, Rockville, Maryland, USA
**Plasmids**		
pDG1661	Source of chloramphenicol resistance gene, Cm^r	Kim and Timmusk (2013)
pMA5	Expression plasmid for *Bacillus*, Amp^r, Kan^r	Liu and Du (2012)
pMA5-*luxS*	*luxS* gene complement and over expression vector by fusing *luxS* into the *Bam*HI/*Hin*dIII restriction site of pMA5	This study
pRN5101	Gene knockout plasmid, thermo-sensitive plasmid, Amp^r, Erm^r	Zhang et al. (2018)
pRN5101-*Cm*	*luxS* gene knockout vector containing two DNA fragments homologous to the	This study

	upstream and downstream regions of the *luxS* and chloramphenicol resistance gene from pDG1661, Cm^r	

Table S2 Primers used in this study

Primer	Sequence (5'-3')	Purpose
*luxS*up-F	GCAAAAGACATAATCGATAAGCTTCGACCT TAATCCAGCGTGTGCCCTCAAAC	To amplify upstream fragment of *luxS* for gene knockout
*luxS*up-R	CTATTGCCGATGATAAGCTGTCAAACATGA GGAGCCTTTACAATCGTGTGGTC	
*luxS*down-F	GGGCTCATGAGCGCTTGTTTCGGCGTGGC ATTAGCCGCGATCCTTTCAAAAGAGTAT	To amplify downstream fragment of *luxS* for gene knockout
*luxS*down-R	CGATGCGTCCGGCGTAGAGGATCCGAGCT GGAGCCATTACTGTCTGTACTTTTAT	
Cm^r-F	GACCACACGATTGTAAAGGCTCCTCATGTT TGACAGCTTATCATCGGCAATAG	To amplify chloramphenicol resistance gene from pDG1661 for gene knockout
Cm^r-R	ATACTCTTTTGAAAGGATCGCGGCTAATGC CACGCCGAAACAAGCGCTCATGAGCCC	
pRN-F	GCAAAAGACATAATCGATAAGCTT	For verification of the construction of pRN5101-*Cm* by PCR or sequencing
pRN-R	ATGCGTCCGGCGTAGAGGATCCAC	
luxS-F	TTATGTAAGAGCAGCGGGAGTAGA	For verification of *P. polymyxa* HY96-2-△*luxS* strain by PCR or sequencing
luxS-R	CAGCCTCCAACACAATCTTCA	
1121-F	CCACCTAAAAAGGAGCGATTTACATATGAT GGCAAAAGTAGAAAGC	To amplify *luxS* for gene complementation and gene over expression
1121-R	GCTTGAGCTCGACTCTAGAGGATCCTTAAA ATACTCTTTTGAAAGGATCGCGGC	
TY-F	GTTGCCGGAAAGAGCGAAAATGCC	For verification of the construction of pMA5-*luxS*, *P. polymyxa* HY96-2-△*luxS::luxS* and *P. polymyxa* HY96-2-*luxS* by PCR or sequencing
TY-R	ACGCGTGATCTCAGATCTGGTACG	
DL*luxS*-F	GACGTATCTGAGAGACGAAATC	To amplify 119 bp fragment of *luxS* for qPCR
DL*luxS*-R	CCAAAGCAAGTGCAATTTCCTC	
27F	AGAGTTTGATCCTGGCTCAG	To amplify V3 region in 16S rRNA gene for qPCR
1492R	GGTTACCTTGTTACGACTT	

Table S3 Chemicals used in this study

Chemicals	Source
Acetone	Hangzhou Gaojing Fine Chemical Co., Ltd. (Hangzhou, Zhejiang, China)
Nutrient solution	Jiangsu Tongqian Ecology Technology Co., Ltd. (Suzhou, Jiangsu, China)
Agar	Shanghai Zhonghe Chemical Technology Co., Ltd. (Shanghai, China)
Sucrose	Shanghai Titanchem Co., Ltd. (Shanghai, China)
Peptone	Sangon Biotech Co., Ltd. (Shanghai, China)
Yeast powder	Sangon Biotech Co., Ltd. (Shanghai, China)
NaCl	Sinopharm Chemical Reagent Co., Ltd. (Shanghai, China)
Mannitol	Sinopharm Chemical Reagent Co., Ltd. (Shanghai, China)
Ethanol	Sinopharm Chemical Reagent Co., Ltd. (Shanghai, China)
Crystal violet	Sinopharm Chemical Reagent Co., Ltd. (Shanghai, China)
Glycerol	Sinopharm Chemical Reagent Co., Ltd. (Shanghai, China)
Sorbitol	Shanghai Dibo Biotechnology Co., Ltd. (Shanghai, China)
K_2HPO_4	Shanghai Lingfeng Chemical Reagent Co., Ltd. (Shanghai, China)
$MgSO_4 \cdot 7H_2O$	Shanghai Lingfeng Chemical Reagent Co., Ltd. (Shanghai, China)
25% Glutaraldehyde	Shanghai Lingfeng Chemical Reagent Co., Ltd. (Shanghai, China)
$NaH_2PO4 \cdot 2H_2O$	Shanghai Lingfeng Chemical Reagent Co., Ltd. (Shanghai, China)
$Na_2HPO4 \cdot 12H_2O$	Shanghai Macklin Biochemical Co., Ltd. (Shanghai, China)

2. Supplementary figures

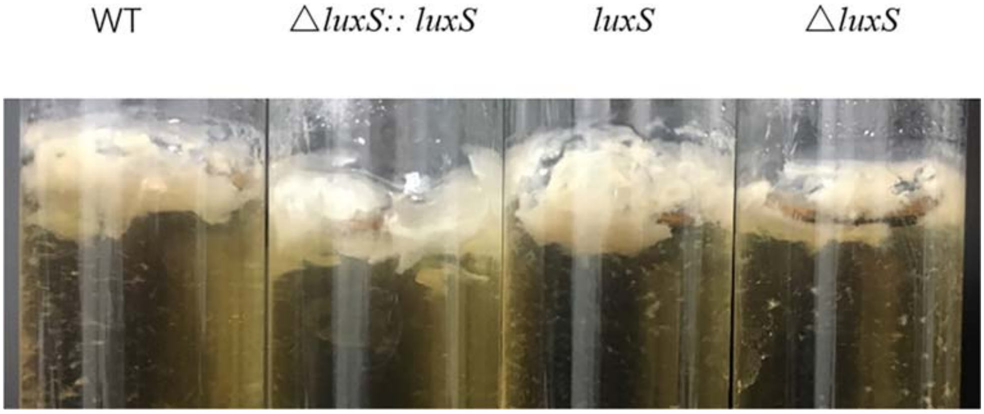

Fig. S1 Images of biofilms of *Paenibacillus polymyxa* HY96-2 and its mutants attached glass tubes on the 20th day post inoculation. WT means *P. polymyxa* HY96-2 wild type strain; △*luxS*:: *luxS* means the *luxS* complement strain, *P. polymyxa*-HY96-2-△*luxS*::*luxS*; *luxS* means the *luxS* over-expression strain, *P. polymyxa*-HY96-2-*luxS*; △*luxS* means the *luxS* deletion strain, *P. polymyxa*-HY96-2-△*luxS*.

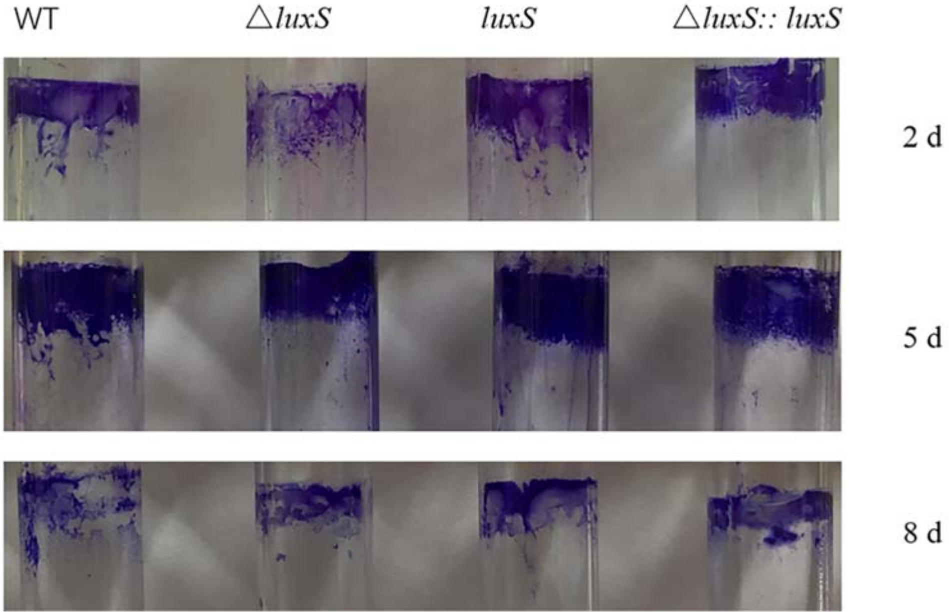

Fig. S2 Images of biofilms of *P. polymyxa* HY96-2 and its mutants attached glass tubes stained by crystal violet. WT means *P. polymyxa* HY96-2 wild type strain; △*luxS* means the *luxS* deletion strain, *P. polymyxa*-HY96-2-△*luxS*; *luxS* means the *luxS* over-expression strain, *P. polymyxa*-HY96-2-*luxS*; △*luxS*:: *luxS* means the *luxS* complement strain, *P. polymyxa*-HY96-2-△*luxS*::*luxS*. 2d, 5d, and 8d mean the 2nd day, 5th day and 8th day post inoculation.

REFERENCES

Kim SB, Timmusk S (2013) A simplified method for gene knockout and direct screening of recombinant clones for application in *Paenibacillus polymyxa*. PLoS One 8(6):1-6. doi:10.1371/journal.pone.0068092

Liu SL, Du K (2012) Enhanced expression of an endoglucanase in *Bacillus subtilis* by using the sucrose-inducible *sacB* promoter and improved properties of the recombinant enzyme. Protein Expression Purif 83(2):164-168. doi:10.1016/j.pep.2012.03.015

Woodcock DM, Crowther PJ, Doherty J, Jefferson S, DeCruz E, Noyer-Weidner M, Smith SS, Michael MZ, Graham MW (1989) Quantitative evaluation of *Escherichia coli* host strains for tolerance to cytosine methylation in plasmid and phage recombinants. Nucleic Acids Res 17: 3469-3478. doi:10.1093/nar/17.9.3469

Zhang L, Cao C, Jiang RF, Xu H, Xue F, Huang WW, Ni H, Gao J (2018) Production of R, R-2,3-butanediol of ultra-high optical purity from *Paenibacillus polymyxa* ZJ-9 using homologous recombination. Bioresour Technol 261:272-278. doi:10.1016/j.biortech.2018.04.036
